# Efficacy of two formulations of afoxolaner (NexGard® and NexGard Spectra®) for the treatment of generalised demodicosis in dogs, in veterinary dermatology referral centers in Europe

**DOI:** 10.1186/s13071-018-3083-2

**Published:** 2018-09-10

**Authors:** Wilfried Lebon, Massimo Beccati, Patrick Bourdeau, Thomas Brement, Vincent Bruet, Agnieszka Cekiera, Odile Crosaz, Céline Darmon, Jacques Guillot, Marion Mosca, Didier Pin, Jaroslaw Popiel, Dorota Pomorska Handwerker, Diane Larsen, Eric Tielemans, Frédéric Beugnet, Lénaïg Halos

**Affiliations:** 1 Boehringer Ingelheim Animal Health, CRSV, 805 Allée des Cyprès, 01150 Saint-Vulbas, France; 2 Boehringer Ingelheim Animal Health, 29 avenue Tony Garnier, 69007 Lyon, France; 3 Centro Medico Veterinario ADDA, Via Roma, 3, 24042 Capriate San Gervasio, Italy; 4 Unité de Dermatologie, Parasitologie des Carnivores et des Equidés, Mycologie, Ecole Vétérinaire de Nantes, Site de la Chantrerie d’Oniris - LUNAM, CS 40706 - 44307, Nantes Cedex 03, France; 5 Wroclaw University of Life Sciences, Faculty of Veterinary Medicine, Pl. Grunwaldzki 47, 50-366 Wrocław, Poland; 6 Unité de Parasitologie, Mycologie, Dermatologie, CHUVA, Ecole Nationale Vétérinaire d’Alfort, 94704 Maisons-Alfort, France; 7 Université de Lyon, VetAgro Sup, Interaction Cellule Environnement, Unité de Dermatologie, 1, Avenue Bourgelat, 69280 Marcy-l’Etoile, France; 8 Lubelska Poliklinika Weterynaryjna, Aleja Kraśnicka 89, 20-718 Lublin, Poland; 9 Boehringer Ingelheim Animal Health, Duluth, GA 30096-4640 USA

**Keywords:** *Demodex canis*, Generalised demodicosis, Mite, Dog, NexGard®, NexGard Spectra®, Afoxolaner, Isoxazoline, Oral, Treatment

## Abstract

**Background:**

A multi-centre field trial was conducted to evaluate the efficacy of afoxolaner based chewables (NexGard® or NexGard Spectra®) for the treatment of generalised demodicosis caused by *Demodex canis* in dogs under field conditions in France, Italy and Poland.

**Methods:**

Client-owned dogs, diagnosed positive for *Demodex* mites by pre-treatment skin scrapings and presenting clinical signs of generalised demodicosis were included. Dogs were orally treated with afoxolaner three times at monthly intervals. Of the 50 dogs enrolled, 48 completed the whole study. Efficacy of the treatments was assessed monthly by *Demodex* mite counts and physical examination with special regard to the severity and extension of skin lesions.

**Results:**

Treatments were well tolerated in all dogs and resulted in a rapid reduction of mites, with all post-treatment mite counts significantly lower than baseline. The number of mites was reduced by 87.6%, 96.5% and 98.1% on Days 28, 56 and 84, respectively. In addition, the skin lesion severity and extent scores as well as the pruritus were all significantly lower at all post-treatment visits compared to the pre-treatment assessment.

**Conclusions:**

This clinical field study demonstrated that monthly administrations of afoxolaner in NexGard® or NexGard Spectra®, offered a convenient and reliable solution for the treatment of canine generalised demodicosis.

## Background

Demodicosis is one of the most frequent skin conditions in dogs. It is a parasitic disease caused by mites of the genus *Demodex* [1, 2]. A small number of mites are usually considered as a normal component of the dog’s skin microbiota, but their proliferation leads to a potentially serious condition [3, 4]. Puppies acquire mites from their mother in the first days of their life through direct skin contact [2]. The evolution from asymptomatic carriage to skin disease may be related to a particular cellular immunodeficiency allowing the multiplication of the mites, although the pathogenesis is not yet elucidated [3–5]. Canine demodicosis is classically divided into two main clinical manifestations, i.e. localised and generalised demodicosis. The localised form appears as patches of alopecia and mild erythema in limited areas of the body, usually in young dogs, although it may also affect older ones. It can regress spontaneously without treatment [2]. Generalised demodicosis is more severe and can even be fatal if a secondary bacterial infection develops [2]. It may evolve from the localised condition or occur spontaneously especially in older animals with underlying diseases [5, 6]. Recently, it has been proposed that localised demodicosis is characterised as no more than four lesions with a diameter of up to 2.5 cm, while canine generalised demodicosis is characterised by five or more affected areas, or by lesions covering an entire region of the body, and/or demodectic podal dermatitis involving two or more paws [3, 4, 6, 7]. In the case of generalised demodicosis, the affected areas are frequently erythematous, with comedones, hair loss, follicular papules to pustules, and scales. Secondary severe bacterial infections are frequent. Suspicion of demodicosis based on clinical signs has to be confirmed by the detection of mites in deep skin scrapings. Alternatively, skin biopsy or hair plucks may also be performed [3, 4, 6].

Generalised demodicosis is a very challenging disease to treat effectively. Only a few drugs and formulations, either topical or systemic, are registered [4, 8]. Many treatment protocols in the field include off label use of macrocyclic lactone, providing variable efficacy with potential for toxicity, especially in dogs carrying *MDR-1* gene mutations [3, 9, 10]. Recently, a new class of insecticides/acaricides, the isoxazolines, demonstrated very good efficacy against many ectoparasites of dogs and cats: fleas and ticks, but also mites, including *Otodectes cynotis*, *Sarcoptes scabiei*, *Lynxacarus radovskyi* and *Demodex canis* [11–18]. Among the isoxazolines, the efficacy of afoxolaner against *D. canis* has been demonstrated in one laboratory study involving naturally infested animals conducted in South Africa. In this study, afoxolaner was administered at fortnighly interval for one month then at a monthly interval for two additional months and demonstrated > 99% reduction in mite counts [11]. The purpose of the present field study was to assess the efficacy of monthly oral administration of afoxolaner in two different formulations, alone (NexGard®) and in combination with milbemycin oxime (NexGard Spectra®) against generalised canine demodicosis in the European pet dog population.

## Methods

### Design

This multi-center field study was held in France, Italy and Poland between January 2016 and March 2017, and was conducted in accordance with Good Clinical Practices as described in the International Cooperation on Harmonization of Technical Requirements for Registration of Veterinary Medicinal Products, VICH Guideline 9 [19].

### Animals

Client-owned dogs of various breeds and of both sexes, weighing at least 2 kg, with a minimum age of 8 weeks and presenting clinical signs of generalised demodicosis were considered eligible for the study.

Criteria for inclusion were the presence of clinical signs of generalised demodicosis (i.e. erythema, hair loss, follicular casts and crust, and/or pyoderma) on 5 or more areas, or pododemodicosis on 2 or more paws and at least 5 live *Demodex* spp. mites (i.e. at least 1 alive mite/alopecic area). All dogs were subjected to a physical examination before treatment to be considered suitable for inclusion into the study. Clinical history and ongoing medications were recorded at inclusion.

### Treatment

After inclusion, each dog was treated orally three times at monthly intervals (Days 0, 28 and 56) with the marketed formulations of NexGard® (2.7 mg/kg afoxolaner) or NexGard Spectra® (2.5 mg/kg afoxolaner and 0.5 mg/kg milbemycin oxime) according the European label instructions. The veterinarian could choose NexGard® or NexGard Spectra® based on the needs of the client, i.e. anthelmintic activity of milbemycin oxime. At least 30% of the dogs were required to be treated with NexGard Spectra®. Dogs were weighed before each treatment for appropriate dose determination.

Treated animals were observed for at least 5 min after each administration to ensure that the chew was swallowed. Personnel involved in the assessment of product efficacy were not blinded to treatment as there was no negative control group, and the primary efficacy variable was the comparison of the mite count with the initial pre-treatment count for each individual dog.

Owners were questioned at each visit about any abnormal observation seen during the study period. Dogs were managed under normal conditions by their owners. Out of the 50 dogs enrolled, 48 completed the study on Day 84.

### Mite counts

Mite counts were performed on Days 0, 28, 56 and 84. Deep skin scrapings were performed in duplicate from five sites with skin lesions on the days of clinical evaluation. Skin scrapings were made of a 2 × 2 cm surface with a blade until capillary oozing occurred. The collected samples were placed onto a microscope slide and mixed with mineral oil and observed under microscope for total mite counts. Live adults, nymphs and larvae as well as dead mites and skeleton were counted. The same sites were scraped at each subsequent examination.

### Clinical scoring

Both severity and extent of clinical signs consistent with generalised demodicosis were evaluated at inclusion and on each day of the mite counts. Five clinical signs were evaluated: alopecia, erythema, papules, pustules and scales/crusts. The severity of the clinical signs was scored as absent (0), mild (1), moderate (2), or severe (3). When present, the extent of the lesion was scored as “limited” [seen on up to 1/3 of the (head + body) surface]; “marked” [seen on up to 2/3 of the (head + body) surface]; and “generalised” [seen all over the (head + body) surface]. In addition, the intensity of pruritus was evaluated by the veterinarian according to a canine pruritus scale [20] and scored from 0 (absent) to 10 (intense).

### Statistical analyses

The statistical analysis was performed using SAS System® v.9.4 (SAS Institute Inc., Cary, NC, USA). For all statistical tests, a nominal significance level of 5% (*P* < 0.05) was applied. No adjustment for multiple tests was performed.

The primary antiparasitic efficacy variable was the reduction of the number of live mites (adults and immature stages) on Day 84 compared to the baseline (pre-treatment). The average percentage reduction in mite counts was calculated using Abott’s formula:

$$ \mathrm{Efficacy}\ \left(\%\mathrm{mite}\ \mathrm{reduction}\right)=100\times \left[\left(\mathrm{C}-\mathrm{T}\right)/\mathrm{C}\right] $$

where C is the arithmetic mean of the baseline count and T is the arithmetic mean of the Day 84 count. The difference between live mite counts on Days 28, 56 and Day 84 *versus* baseline was tested using a signed rank test.

In addition, the difference between percentage reductions in mite counts in two different classes of age (dogs younger than 18 months and dogs older than 18 months) was tested using a Wilcoxon Rank Sum test with continuity correction.

The secondary variable was the resolution of clinical signs. Lesion severity and extent scores were recorded for each dog at each time-point for each lesion (alopecia, erythema, pustules, papules and scales/crusts). The total skin lesion and total extent of the lesions were calculated for each dog as the sum of the skin lesions scores and extent of the lesions scores, respectively. These scores, as well as the pruritus score, were summarized by time-points. Differences in scores between Days 28, 56, 84, and baseline were tested using a Cochran-Mantel-Haenszel (CMH) test [(*F*) Mean Score Difference test].

## Results

### Inclusion

A total of 50 dogs (29 females and 21 males) weighing from 2.4 to 46.0 kg were enrolled in the study (14 dogs from France, 21 dogs from Italy and 15 dogs from Poland). Forty-four dogs were pure breed and only six were mixed breeds (Table 1). The most prevalent breeds enrolled were American Staffordshire Terrier (10.2%), English Bulldog (10.2%), French Bulldog (8.2%), Pug (8.2%) and Yorkshire Terrier (8.2%). The dogs were 3 months to 15 years-old. Twenty-seven of them were younger than 18 months while 23 dogs were older. Little information was available on concomitant diseases at inclusion. One 6-year-old French Bulldog was reported with Cushing syndrome, one 2-year-old crossbreed Maremma Sheepdog had leishmaniosis and one 15-year-old poodle was displaying polyuria-polydipsia associated with breast neoplasia.

**Table 1:** Summary of dog information and clinical history when available. The total mite counts at enrolment (Day 0) and study end (Day 84) is indicated

Dog name	Age	Breed	Sex	Lesions at enrolment and clinical history	Mite count	
					Day 0	Day 84
Cherry	3 months	Pug	F	Alopecia, erythema, papules, pustules and scaling	182	0
Kenzo	4 months	Chihuahua	M	Severe alopecia and papules	213	0
Liner	6 months	Staffordshire Bull Terrier	M	Alopecia, erythema, papules, pustules and scaling	140	0
Mia	6 months	Crossbreed Pinscher	F	Multifocal alopecia without pruritus	18	0
Figa	6 months	Whippet	F	Severe alopecia and erythema	44	11
Loca	7 months	French Bulldog	F	Alopecia, erythema and scaling	2163	0
Shaya	9 months	American Staffordshire Terrier	F	Moderate lesions of demodicosis	43	0
Loki	9 months	Bull Terrier	M	Severe lesions of demodicosis	121	0
Jagoda	9 months	English Bulldog	F	Alopecia, erythema and scaling	77	5
Kaya	11 months	America Staffordshire Terrier	F	Severe alopecia and erythema	110	0
Baby	10 months	Crossbreed German Shepherd	F	Alopecia, papules and pustules	31	0
Zoe	10 months	Boston Terrier	F	Multifocal alopecia	41	0
Kora	10 months	Drathaar	F	Alopecia, erythema and scaling	46	0
Ares	11 months	Yorkshire Terrier	M	Severe demodectic pododermatitis	57	3
Luna	11 months	American Staffordshire Terrier	F	Severe demodectic pododermatitis	386	0
Hollywood	5 months	American Staffordshire Terrier	F	Multifocal alopecia with pruritus since 1 month	39	0
Borys	5 months	Beagle	M	Demodectic pododermatitis	49	0
Mya	7 months	Crossbreed Dogo Argentino	F	Multifocal alopecia	42	0
Odi	8 months	Mongrel	M	Demodectic pododermatitis	40	0
Elsa	1 year	Pug	F	Severe alopecia	13	0
Argo	1 year	Dobermann	M	Alopecia and scaling; demodicosis diagnosed 6 months earlier and treated with amitraz	97	0
Ares	1 year	American Staffordshire Terrier	M	Alopecia, erythema, papules pustules and scaling	36	0
Klops	1.1 year	Pug	M	Severe alopecia erythema, papules, pustules and scaling	2349	0
Achille	1.2 year	Pitbull	M	Alopecia and scaling	53	0
Buch	1.5 year	German Shepherd	M	Alopecia, erythema, papules, pustules	18	NCA
Laure	2 years	Pointer	F	Severe erythema and moderate alopecia for one month	77	0
Benek	2 years	English Bulldog	M	Severe demodectic pododermatitis	97	9
Lili	2 years	Yorkshire Terrier	F	Demodectic pododermatitis	56	0
Ibex	3 years	Jack Russell Terrier	F	Moderate alopecia and erythema	36	0
Kenzo	3 years	Basset Bleu de Gascogne	F	Alopecia, erythema, pustules	256	0
Carlitos	4 years	Pug	M	Severe lesions of chronic demodectic pododermatitis	25	0
Gruby	4 years	English Bulldog	M	Demodectic pododermatitis	476	0
Buza	5 years	English Bulldog	F	Severe alopecia and erythema	69	6
Brego	5 year	White Swiss Shepherd	M	Alopecia, erythema and scaling	39	0
Meggy	8 years	Yorkshire Terrier	F	Demodectic pododermatitis	64	2
Szajba	8 years	Toy Schnauzer	F	Alopecia and scaling	51	0
Hector	1 year	French Bulldog	M	Severe alopecia, erythema and scaling	100	0
Nari	1 year	Podenca	F	Severe alopecia and erythema for 2 months	274	0
Jazzie	1.5 year	French Bulldog	F	Alopecia, erythema, pustules	213	0
Sonia	10 year	Mongrel	F	Severe demodectic pododermatitis	46	4
Angy	10 year	Yorkshire Terrier	M	Alopecia, erythema and scaling	18	0
Lilly	15 years	Poodle	F	Demodectic pododermatitis, polyuria, polydipsia, breast neoplasia, heart failure	239	NCA
Cosmo	2 years	Crossbreed Maremma Sheepdog	M	Severe alopecia, papule, pustules and pruritus; leishmaniosis	77	29
Jacky	3 years	Jack Russell Terrier	F	Demodectic pododermatitis	22	0
Shelly	4 years	Maltese	F	Severe alopecia, erythema and scaling	62	3
Moira	4 years	Pinscher	F	Atopic dog with alopecia and scaling	18	0
Asia	5 years	English Bulldog	F	Alopecia, erythema and scaling	93	0
Leon	6 years	French Bulldog	M	Cushing syndrome and severe demodectic pododermatitis	92	53
Rocky	7 years	Labrador Retriever	M	Severe demodectic pododermatitis	30	8
Hoffman	8 years	Pitbull	M	Demodectic pododermatitis	211	37

*Abbreviations: F* female, *M* male, *NCA* no count available

Thirty-one dogs were treated with NexGard® and 19 dogs with NexGard Spectra®.

### Mite counts

All dogs were confirmed to have more than five live *Demodex* mites before treatment with an arithmetic mean count of 183 mites/dog (range of 13–2349). Treatment with afoxolaner resulted in a rapid and significant reduction of the number of mites in all post-treatment counts (Table 2). Overall, afoxolaner miticidal efficacy was shown to be 87.6%, 96.5% and 98.1% on Days 28, 56 and 84, respectively. At the end of the study, 75% of the dogs had no live mites. At this last time-point, the 12 dogs with a positive skin scraping had an arithmetic mean of 3.54 mites.

**Table 2:** *Demodex canis* mite count reduction in dogs treated three times at a monthly interval with oral afoxolaner

	Day 0	Day 28	Day 56	Day 84
Total number of dogs (*n*)	50	50	49	48
Mean mite count (*n*)	183	22.8	6.4	3.5
Count range (*n*)	13–2349	0–191	0–65	0–53
Reduction (%)	na	87.6	96.5	98.1
Mite-free dogs (%)^a^ (no. of mite-free dogs/total no. of dogs)	na	12 (6/50)	38.8 (19/49)	62.5 (30/48)
Signed rank (S)	na	-599	-612	-588
Degrees of freedom	na	48	48	47
*P*-value	na	<0.0001	<0.0001	<0.0001
NexGard-treated animals				
Number of dogs (*n*)	31	31	30	29
Mean mite count (*n*)	229.8	26.5	7.4	4.1
Count range (*n*)	18–2349	0–191	0–65	0–53
Reduction (%)	na	88.5	96.8	98.2
Signed rank (S)	na	-224	-232.5	-217.5
Degrees of freedom		29	29	28
*P-*value	na	<0.0001	<0.0001	<0.0001
NexGard Spectra-treated animals				
Number of dogs (*n*)	19	19	19	19
Mean mite count (*n*)	106.6	16.8	4.8	2.7
Count range (*n*)	13–386	0–91	0–26	0–37
Reduction (%)	na	84.3	95.5	97.5
Signed rank (S)	na	-95	-95	-95
Degrees of freedom	na	18	18	18
*P-*value	na	<0.0001	<0.0001	<0.0001

*Abbreviation*: *na* not applicable

^a^ Mite-free dogs: absence of mite (live or dead) at count

Specific analyses of the efficacy for juvenile (< 18 months) or adult (> 18 months) onset of demodicosis were conducted (Table 3), including or excluding dogs with demodectic podal dermatitis. A significant difference was observed between the efficacy in the dogs younger than 18 months compared to the dogs older than 18 months in the overall treated population (*Z* = 375.5, *P =* 0.018), while no significant difference was observed between the same classes of age if dogs with demodectic podal dermatitis are excluded (*Z* = 375.5, *P =* 0.23).

**Table 3:** Percent efficacy of afoxolaner against *Demodex* spp. according to the age of the dogs and the presence of specific lesions of demodectic pododermatitis

	Dog age		Wilcoxon Rank Sum test	
	< 18 months	> 18 months	*Z*-value	*P-*value
Efficacy (%) against *Demodex* spp. in the overall treated population (*n*)	98.6 (26)	92.1 (22)	375.5	0.018
Efficacy against *Demodex* spp*.* excluding demodectic pododermatitis (*n*)	98.6 (22)	95.7 (12)	153	0.230

*Abbreviation*: *n* number of dogs

### Clinical scores

In order to evaluate the effects of afoxolaner on the clinical expression of demodicosis, all dogs that had received concomitant medications for the control of skin conditions (e.g. antibiotics, corticosteroids, antihistamines) were excluded from the clinical score analyses. Among the 17 excluded dogs, 8 were from the Nexgard® group and 9 from the NexGard Spectra® group. Treatments included chlorhexidine shampoos (9/17), systemic antibiotherapy (6/17) with cephalosporins or fluoroquinolone, oclacitinib (1/17) and food supplementation for immune system activation (beta-glucan) (2/17).

Alopecia and erythema were the two most frequent clinical signs affecting the enrolled animals with 100 and 88%, respectively, of the dogs harboring them (almost half of these dogs presented severe lesions). At the end of the study, 78.1 and 87.5% of the dogs had no alopecia or no erythema, respectively. Total skin lesion score and total extent score and pruritus score were significantly lower on Days 28, 56 and 84 compared to pre-treatment (Day 0) values (Table 4). The evaluation of the prevalence of the individual lesion scores (severity) and the extent score for each of the five selected clinical signs (alopecia, erythema, papules, pustules and scaling) between Day 0 and Day 84 is presented in Table 5.

**Table 4:** Cochran-Mantel-Haenszel (CMH) Mean Score Difference (*F*) test for the total skin lesions score, the total extent of the lesion score and the pruritus score at Day 28, Day 56 and Day 84 compared to Day 0 for 31^a^ dogs treated with afoxolaner who didn’t received concomitant medications for the control of skin conditions

		Day 28	Day 56	Day 84
Total skin lesion score	CMH row mean scores differ	*F*_(1, 62)_ = 19.0	*F*_(1, 63)_ = 38.2	*F*_(1, 62)_ = 45.3
	*P-*value	<0.0001	<0.0001	<0.0001
Total lesion extent score	CMH row mean scores differ	*F*_(1, 62)_ = 15.0	*F*_(1, 63)_ = 33.0	*F*_(1, 62)_ = 45.4
	*P-*value	<0.0001	<0.0001	<0.0001
Pruritus score	CMH row mean scores differ	*F*_(1, 63)_ = 13.7	*F*_(1, 64)_ = 27.6	*F*_(1, 63)_ = 32.2
	*P-*value	0.0002	<0.0001	<0.0001

^a^17 animals were excluded from the analyses because of concomitant medications for the control of skin conditions

**Table 5:** Individual lesion and extent scores (Day 0 and Day 84) for 31 dogs treated with afoxolaner who didn’t received concomitant medications for the control of skin conditions

Lesion	Severity			Extent		
		Day 0	Day 84		Day 0	Day 84
Alopecia	Absent (%)	0	78.1	Absent (%)	0	78.1
	Mild (%)	18.2	18.8	Limited (%)^a^	54.5	21.9
	Moderate (%)	33.3	3.1	Marked (%)^b^	39.4	0
	Severe (%)	48.5	0	Generalised (%)^c^	6.1	0
	CMH row mean scores differ	*F*_(1, 63)_ = 46.7; *P* <0.0001		CMH row mean scores differ	*F*_(1, 63)_ = 42.3; *P* <0.0001	
Erythema	Absent (%)	12.1	87.5	Absent (%)	12.1	87.5
	Mild (%)	18.2	9.4	Limited (%)^a^	51.5	12.5
	Moderate (%)	24.2	3.1	Marked (%)^b^	27.3	0
	Severe (%)	45.5	0	Generalised (%)^c^	9.1	0
	CMH row mean scores differ	*F*_(1, 63)_ = 38.1; *P* <0.0001		CMH row mean scores differ	*F*_(1, 63)_ = 36.3; *P* <0.0001	
Papules	Absent (%)	53.1	93.8	Absent (%)	53.1	93.8
	Mild (%)	12.5	3.1	Limited (%)^a^	25.0	6.3
	Moderate (%)	12.5	3.1	Marked (%)^b^	21.9	0
	Severe (%)	21.9	0	Generalised (%)^c^	0	0
	CMH row mean scores differ	*F*_(1, 62)_ = 13.8; *P* = 0.0002		CMH row mean scores differ	*F*_(1, 62)_ = 13.9; *P* = 0.0001	
Pustules	Absent (%)	56.3	96.9	Absent (%)	56.3	96.9
	Mild (%)	12.5	0	Limited (%)^a^	21.9	3.1
	Moderate (%)	18.8	3.1	Marked (%)^b^	21.9	0
	Severe (%)	12.5	0	Generalised (%)^c^	0	0
	CMH row mean scores differ	*F*_(1, 62)_ = 14.2; *P* = 0.0002		CMH row mean scores differ	*F*_(1, 62)_ = 14.7; *P* = 0.0001	
Scaling crusts	Absent (%)	39.4	96.9	Absent (%)	21.2	93.8
	Mild (%)	15.2	3.1	Limited (%)^a^	51.5	6.3
	Moderate (%)	24.2	0	Marked (%)^b^	21.2	0
	Severe (%)	0	0	Generalised (%)^c^	6.1	0
	CMH row mean scores differ	*F*_(1, 63)_ = 32.1; *P* <0.0001		CMH row mean scores differ	*F*_(1, 63)_ = 33.5; *P* <0.0001	

*Note*: 17 animals were excluded from the analyses because of concomitant medications for the control of skin conditions

^a^Seen on 1/3 of the (head + body) surface

^b^Seen on 2/3 of the (head + body) surface

^c^Seen all over the head + body

**Fig. 1:**
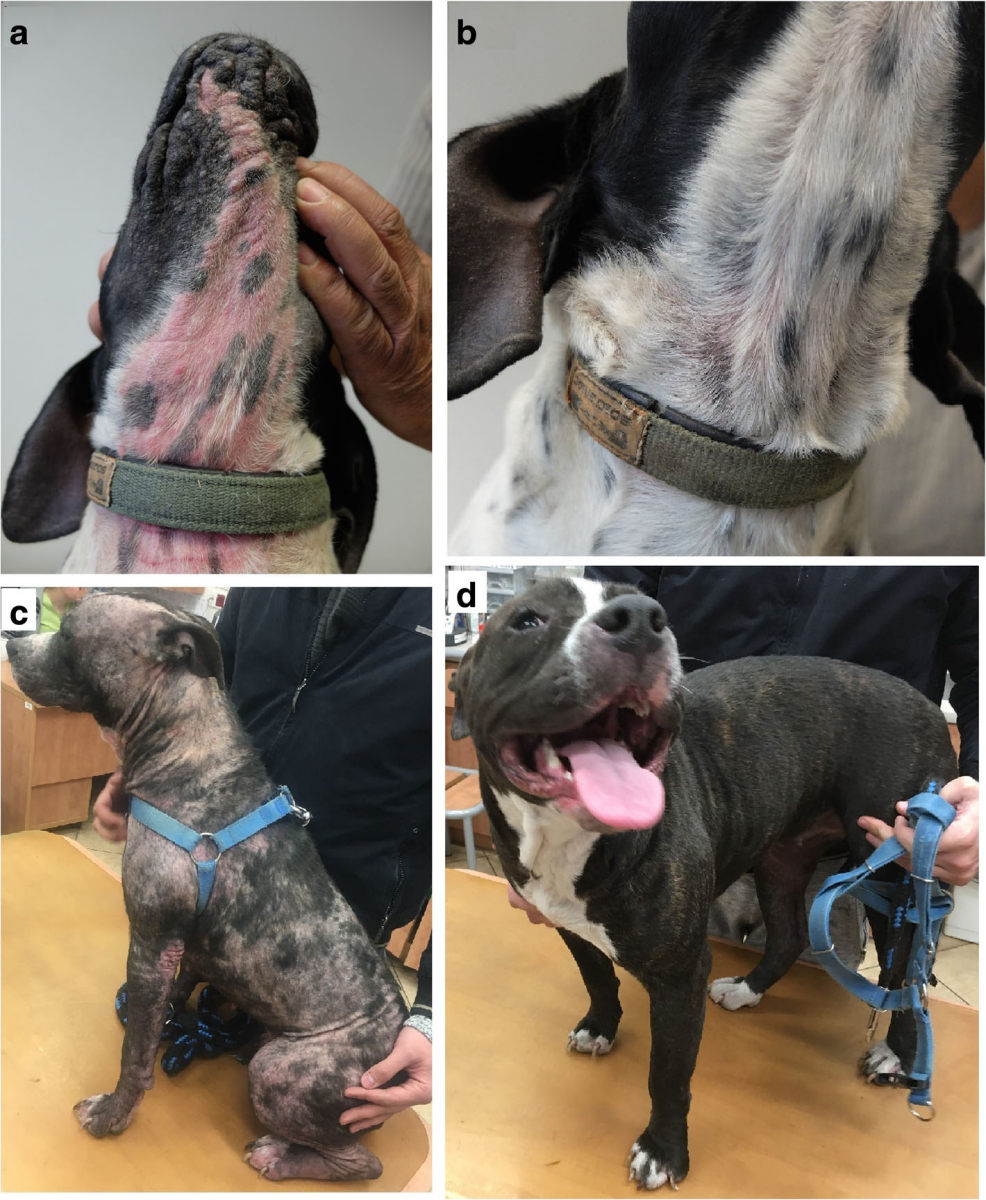
Clinical evolution after monthly administrations of afoxolaner in two dogs with generalised demodicosis. **a** Pre-treatment lesions of erythema and alopecia in a 2-year-old Pointer. **b** Lesions after two treatments with afoxolaner (NexGard®) at a monthly interval (D56). **c** Pre-treatment lesions of multifocal alopecia and erythema in an 11-month-old American Staffordshire Terrier. **d** Lesions after two treatments with afoxolaner (NexGard Spectra®) at a monthly interval (D56)

Afoxolaner administration was thus associated with significantly lower clinical sign scores, lesion extent and pruritus score compared to Day 0 over the course of the treatment (Fig. 1).

### Safety

Except for one dog vomiting a few hours after the first administration, no treatment related adverse event was observed in any dog. One dog from the Nexgard® group was removed at the owner’s request because of an aggressive behavior that jeopardized the appropriate follow-up of the dog, and another dog from the same group previously diagnosed with cancer and chronic heart problems died from heart failure.

## Discussion

This multi-center clinical field study demonstrated that monthly oral administrations of afoxolaner, both alone or in combination with milbemycin oxime, provided a rapid and significant reduction of the number of *Demodex* mites and of clinical signs of demodicosis in privately-owned dogs in Europe. The results obtained are consistent with the findings reported previously with afoxolaner [11] or other isoxazolines [14, 15, 21–23].

No comparison between NexGard® and NexGard Spectra® was performed because the objective was the evaluation of afoxolaner activity independently of the formulation. It was assumed that the addition of milbemycin oxime would have no impact on the overall efficacy of afoxolaner against *Demodex* spp. mites. Indeed, the half-life of milbemycin oxime is very short (2–3 days) with no accumulation. A monthly dose of 0.5 mg/kg of milbemycin oxime would not provide a sufficient amount of active ingredient to improve the control of the disease.

The enrolled dog population reflected the profile of dogs usually presented for demodicosis in veterinary practices. A recent broad-spectrum survey conducted on a cohort of 431 dogs in California identified the Pitbull Terrier group (including American Staffordshire Terrier) as probably predisposed to demodicosis [24] and this was also the most frequent breed group enrolled in the present study. Differentiation between juvenile- and adult-onset demodicosis is sometimes difficult. It is mainly driven by the presence of underlying conditions to manage in addition to the parasitic infestation in adult animals [2, 24]. For this reason, the treatment is often considered easier in younger dogs than in adults. In the present study, 27 dogs were younger than 18 months while 23 dogs were older. The efficacy on Day 84 was 98.6% for dogs under 18 months and 92.1% for older dogs, suggesting that afoxolaner can be used to treat all clinical types of demodicosis. The difference between the efficacies in the two classes of age is significant. This is in accordance with the difference in the course of the disease of adult-onset compared to juvenile-onset of demodicosis well described in the literature.

Out of the 48 dogs which completed the study, 14 had demodectic podal dermatitis. Seven of these dogs were among the dogs still harboring live mites at the end of the study. Demodectic podal dermatitis is more difficult to cure and the prognosis presupposes a longer course of treatment [25]. In addition, demodectic pododermatitis is often related to dogs affected with underlying factors (diabetes mellitus, cancer, strong immunosuppression), which may need continuous protection against *Demodex* spp. proliferation [2]. Interestingly, if dogs with demodectic podal dermatitis are excluded from the analysis comparing classes of age, no significant difference is observed between dogs older or younger than 18 months. This finding corroborates the difficulty of controlling demodectic pododermatitis.

The challenging question that remains is related to the duration of treatment. It is known to be highly variable and depending on individual features. In the American cohort study, juvenile demodicosis was treated within 4.5 months (range 0.25–15) for 86.4% of the dogs. Adult demodicosis was treated within 5.9 months (range 1–24) for 87.7% of the dogs [24]. In veterinary practices, the treatment is stopped after complete remission of clinical signs and two negative skin scrapings performed at a monthly interval [26]. However, according to some authors, dogs should not be considered cured unless no relapse occurs in the year following the end of the specific treatment [2]. In the present study, 19 dogs (40%) had two consecutive negative skin scrapings at Day 56 and Day 84.

Long term compliance is a key factor for the control of chronic diseases [27]. A treatment administrated at a monthly interval is in-line with the monthly follow up of the mite infestation and is expected to improve adherence to treatment.

The need for flea and tick prevention justifies long-term isoxazoline treatment and may prevent relapse/recurrence of demodicosis or even decrease the overall frequency of the disease [24]. It would be of interest to assess the preventive efficacy of these molecules in young dogs predisposed to demodicosis. A long term epidemiological survey of breeds predisposed to demodicosis might help answering this question.

One hypothesis would be that the acaricidal efficacy of isoxazolines used for a sufficient period would eventually kill the whole population of *Demodex* spp. mites present on the body surface of a dog. In that case, no relapse would occur even in the context of demodicosis related to underlying conditions. A recent publication indicated that treatment with isoxazoline (afoxolaner or fluralaner) had a limited effect on cutaneous *Demodex* spp. populations of normal dogs over a 90 day period and thus does not eliminate the mite population on a dog. However, this study was based on a DNA detection using simple PCR with no quantification methods or evaluation of the viability of the mites [28]. Those results should therefore be considered as not conclusive and additional studies are necessary to better understand the effect of isoxazoline on *Demodex* mite populations.

## Conclusions

The high level of activity against *Demodex* spp. achieved with afoxolaner-based products offers new opportunities to veterinarians for the control of demodicosis. It provides new solutions combining safety, efficacy and ease-of-use for improved owner compliance. The potential of choosing a combination product including a nematodicide molecule allows veterinarians to adapt the treatment of demodicosis to specific epidemiological situations such as those encountered in heartworm or lungworm disease enzootic areas.
